# A role for PKD1 in insulin secretion downstream of P2Y_1_ receptor activation in mouse and human islets

**DOI:** 10.14814/phy2.14250

**Published:** 2019-10-07

**Authors:** Shara Khan, Mourad Ferdaoussi, Austin Bautista, Valérie Bergeron, Nancy Smith, Vincent Poitout, Patrick E. MacDonald

**Affiliations:** ^1^ Department of Pharmacology and Alberta Diabetes Institute University of Alberta Edmonton Alberta Canada; ^2^ Département de Médecine Université de Montréal Montréal Quebec Canada; ^3^ Centre de Recherche du Centre Hospitalier de l’Université de Montréal (CRCHUM) Montréal Quebec Canada

**Keywords:** ATP, BMI, insulin, P2Y1, PKD1, *β* cells

## Abstract

Along with insulin, *β*‐cells co‐secrete the neurotransmitter ATP which acts as a positive autocrine signal via P2Y_1_ receptors to activate phospholipase C and increase the production of diacylglycerol (DAG). However, the downstream signaling that couples P2Y_1_ activation to insulin secretion remains to be fully elucidated. Since DAG activates protein kinase D1 (PKD1) to potentiate glucose‐stimulated insulin release, we hypothesized that autocrine ATP signaling activates downstream PKD1 to regulate insulin secretion. Indeed, we find that the P2Y_1_ receptor agonists, MRS2365 and ATP induce, PKD1 phosphorylation at serine 916 in mouse islets. Similarly, direct depolarization of islets by KCl caused PKD1 activation, which is reduced upon P2Y_1_ antagonism. Potentiation of insulin secretion by P2Y_1_ activation was lost from PKD1^−/−^ mouse islets, and knockdown of PKD1 reduced the ability of P2Y_1_ activation to facilitate exocytosis in single mouse *β*‐cells. Finally, qPCR analysis confirmed PKD1 transcript (*PRKD1*) expression in human islets, and insulin secretion assays showed that inhibition of either P2Y_1_ or PKD1 signaling impaired glucose‐stimulated insulin secretion. Human islets showed donor‐to‐donor variation in their responses to both P2Y_1_ and PKD1 inhibition, however, and we find that the P2Y_1_‐PKD1 pathway contributes a substantially greater proportion of insulin secretion from islets of overweight and obese donors. Thus, PKD1 promotes increased insulin secretion, likely mediating an autocrine ATP effect via P2Y_1_ receptor activation which may be more important in islets of donors who are overweight or obese.

## Introduction

Diabetes results from impaired or insufficient insulin secretion from pancreatic islets of Langerhans. While plasma glucose is a key regulator, insulin release is modulated by other nutrients, circulating hormones, the autonomic nervous system, and local paracrine and autocrine signals. The neurotransmitter adenosine triposphate (ATP) is released from *β* cells and acts as a positive autocrine signal by activating P2Y_1_ receptors (Wuttke et al. 2013; Khan et al. 2014). While it has been shown that ATP feedback activates purinergic P2Y_1_ receptors, resulting in activation of phospholipase C (PLC) and spatially restricted production of diacylglycerol (DAG) (Wuttke et al. 2013), the downstream signaling that couples P2Y_1_ activation to enhance insulin secretion remains to be fully elucidated. The elevated plasma membrane DAG concentration locally and transiently activates protein kinase C (PKC) and/or other effectors (possibly PKD1) to potentiate the insulin secretory response (Wuttke et al. 2013; 2016). It has also been demonstrated that generation of DAG leads to activation of protein kinase D1 (PKD1), F‐actin depolymerization, and potentiation of glucose‐stimulated insulin secretion (Ferdaoussi et al. 2012). The first evidence of a role for PKD1 in pancreatic *β*‐cells was provided by Sumara et al. (2009) who showed that PKD1 promoted both insulin secretion and *β*‐cell survival. PKD1 is also involved in the augmentation of insulin secretion via muscarinic M3 receptor signaling (Kong et al. 2010) and affects the second phase of glucose‐stimulated insulin secretion through the PPAR*β*/*δ* pathway (Iglesias et al. 2012). In regulated insulin secretion, PKD1 and its substrate Arfaptin‐1 promote insulin vesicle fission at the trans‐Golgi network (Gehart et al. 2012). Recent work on pancreatic *β* cell‐specific PKD1 deletion showed, however, that while insulin secretion was not impaired in chow‐fed mice, an insulin secretory deficit was evident following high‐fat feeding (Bergeron et al. 2017). This raises the possibility that PKD1 signaling within *β* cells may be particularly important in maintaining secretory function under metabolic stress.

PKD1 is a serine/threonine kinase that belongs to the Ca^2+^/calmodulin‐dependent kinases (CaMKs) superfamily (Valverde et al. 1994). Its activation is dependent in part on the phosphorylation of two activation loop sites, serine 744 and serine 748, via a PKC‐dependent signaling pathway (Waldron and Rozengurt, 2003). In addition, serine 916 has been identified as an autophosphorylation site indicative of PKD activation (Matthews et al. 1999). However, in humans, the autophosphorylation site for PKD1 is serine 910 (Nishikawa et al. 1997). While we previously showed that ATP acts as a positive autocrine signal in human *β* cells by activating P2Y_1_ receptors, stimulating electrical activity, and increasing [Ca^2+^]_i_ by stimulating Ca^2+^ influx and evoking Ca^2+^ release via InsP_3_‐receptors in the endoplasmic reticulum (Khan et al. 2014), here we investigate the other arm of the PLC pathway mediated by DAG‐induced PKD1 activation. We find that ATP signaling via P2Y_1_ activates PKD1 downstream and potentiates *β* cell exocytosis. Taken together with our previous work (Khan et al. 2014), this suggests that an ATP‐PKD1 axis works in concert with the InsP_3_‐receptor‐dependent Ca^2+^ store mechanism to enhance insulin secretion in response to glucose. This pathway appears particularly important in islets from obese but nondiabetic human donors since the ability of P2Y_1_ antagonism to suppress insulin secretion correlates with donor body mass index (BMI), and a similar trend is observed for PKD1 inhibition. This, together with recent studies in high‐fat fed mice lacking *β* cell PKD1, suggests a role for the pathway in increased insulin secretory responses observed in conditions of metabolic stress.

## Methods

### Cells and cell culture

Islets from male C57Bl/6 or from mice of both sexes lacking *β* cell PKD1, described in detail previously (Bergeron et al. 2017), were isolated by collagenase digestion and cultured in RPMI 1640 containing 11.1 mmol/L glucose with 10% FBS and 100 U/mL of penicillin/streptomycin. *β*PKD1KO and MIP‐CreERT mouse islets were described previously (Bergeron et al. 2017). Human islets were isolated from donor pancreata at the Alberta Diabetes Institute IsletCore (http://www.isletcore.ca) at the University of Alberta (Edmonton, Alberta, Canada) or the Clinical Islet Laboratory at the University of Alberta and were cultured in low‐glucose (5.5 mmol/L) DMEM with l‐glutamine, 110 mg/L sodium pyruvate, 10% FBS, and 100 units/mL penicillin/streptomycin. In total, islets from 16 human donors were examined in this study. For single‐cell experiments, mouse islets were dispersed by shaking in cell dissociation buffer (Gibco, Thermo Scientific) and plated in 35‐mm culture dishes. Islets and dispersed cells were cultured at 37°C and 5% CO_2_. INS 832/13 cells (from C. Newgard, Duke University, Durham, NC) were cultured in RPMI 1640 containing 11.1 mmol/L glucose with 10% FBS, 10 mmol/L HEPES, 0.29 mg/mL l‐glutamine, 1 mmol/L sodium pyruvate, 50‐*μ*L *β*‐mercaptoethanol, and 100 U/mL of penicillin/streptomycin. Islets, INS 832/13 cells, or dispersed cells were cultured at 37°C and 5% CO_2_.

### Immunoblotting

Mouse islets were preincubated for 1 h in 1 mmol/L glucose Krebs‐Ringer Bicarbonate (KRB) and subsequently treated for 20 min with P2Y_1_ agonists, ATP (10 *μ*mol/L) and MRS2365 (100 nmol/L; EC_50_ 0.4 nmol), or 30‐mmol KCl ± P2Y_1_ antagonist, MRS2500 (1 *μ*mol/L; EC_50_ 0.78 nmol). Following treatment, cells were lysed in buffer containing (in mmol/L): 20 Tris‐HCl (pH = 7.5); 150 NaCl, 1 EDTA, 1 EGTA, 2.5 sodium pyrophosphate, 1 EDTA, 1 b‐glycerophosphate, 25 N‐ethylmaleimide, 1% Triton X‐100, and 1X protease inhibitor cocktail. Protein lysates were separated using sodium dodecyl sulfate‐polyacrylamide gel electrophoresis (SDS‐PAGE), transferred to nitrocellulose membranes, incubated overnight at 4°C with primary antibodies (phospho‐PKD/PKCmu (serine 916) Rabbit A [Cell signalling] and anti‐*β*‐actin; sc‐4778 [Santa Cruz Biotechnology]), and visualized with horseradish peroxidase‐labeled anti‐rabbit IgG as secondary antibodies (Amersham, Baie d’Urfe, PQ). Images were acquired using a ChemiDoc MP System (Bio‐Rad) and analyzed using the densitometry analysis function in Image Lab Software 5.2.1 (Bio‐Rad) and normalized to actin as a loading control and expressed as a fold increase from baseline.

### Static incubation insulin secretion

For static incubations, measurements were performed at 37°C in KRB solution containing (in mmol/L): 115 NaCl, 5 KCl, 24 NaHCO_3_, 2.5 CaCl_2_, 1 MgCl_2_, 10 HEPES, and 0.1% fatty‐acid free BSA (pH 7.4). Intact mouse or human islets in batches of 15 islets each were preincubated for 2 h at 1.0 mmol/L glucose‐KRB. Islets were transferred to fresh KRB solution containing 1.0 mmol/L glucose for 1 h, followed by incubation for 1 h in 10.0 mmol/L glucose‐KRB. Each condition was run in triplicate. Supernatant fractions were collected, and islets were lysed in buffer containing 1.5% concentrated hydrochloric acid, 23.5% acetic acid, and 75% ethanol for assay of insulin content. Samples were stored at –20°C and assayed for insulin via insulin detection kits (Meso Scale Discovery).

### Electrophysiology

Dispersed mouse islets were plated in 35‐mm culture dishes. Prior to electrophysiological recordings, mouse pancreatic islet cells were transfected with scrambled or PKD1 siRNA for 48 h. Transfected cells were identified by labeling with Alexa Fluor 488 dye. *β* cells were identified by insulin immunostaining following the experiment as previously described (Khan et al. 2014). Solutions used for capacitance measurements have been previously described (Khan et al. 2014). The P2Y_1_ agonist, MRS2365 (100 nmol/L), was added to the extracellular bath solution for the duration of the recordings. One group of cells were patched following a 15‐min pretreatment with thapsigargin (10 *μ*mol/L). The standard whole‐cell technique with the sine + DC lockin function of an EPC10 amplifier and Patchmaster software (HEKA Electronics, Lambrecht/Pfalz) was used. Experiments were performed at 32°C–35°C. Quantification of the average cumulative increase in the capacitance of 500‐msec depolarizations from –70 to 0 mV was calculated.

### siRNA constructs and quantitative PCR

PKD1 and scrambled siRNA constructs were from Origene. *Prkd1 Mouse siRNA Oligo Duplex (Locus ID 18760), CAT#: SR421194 *(https://www.ncbi.nlm.nih.gov/nuccore/NM_008858). These were transfected in dissociated mouse islet cells using DharmaFECT 1 (GE Healthcare, Mississauga, ON). For quantitative PCR, RNA from dispersed mouse islets was extracted 48‐h posttransfection from aliquots of 150 islets using TRIzol reagent (Life Technologies, Burlington, ON), and cDNA was synthesized using Super Script II and oligodT (Life Technologies) according to the manufacturer's protocol. Real‐time PCR to detect PKD1 was performed as previously described (Ferdaoussi et al. 2012) *using the Faststart DNA Master PLUS SYBR Green Kit (Roche). Primers for mouse Prkd1 gene (Fwd 5′‐TAGCCAAGGGTGACTCAAGG‐3′ and Rev 5′‐ CTGGACATGTGGTCTGTTGG‐3′) or β‐actin (Fwd 5′‐TGAAGTGTGACGTTGACATCC‐3′ and Rev 5′‐ACAGTGAGGCCAGGATAGAGC‐3′) were designed using Primer3. Results are expressed as the ratio of target mRNA to β‐actin mRNA. Quantitative PCR was also used to measure PKD1 mRNA expression in human embryonic kidney cells and in isolated human islets from healthy donor (n* = *3 replicates from one donor). Primers were as follows: (Fwd 5′‐TTCTCCCACCTCAGGTCATC‐3′ and Rev:5′‐ CCAAATCCCTGGAAGGAAAT‐3′).* Quantitative PCR was also used to measure *PRKD1* mRNA expression in human embryonic kidney cells and in isolated human islets from healthy donor (*n* = 3 replicates from one donor). Primers were as follows: left: TTCTCCCACCTCAGGTCATC and right: CCAAATCCCTGGAAGGAAAT.

### Ethics approvals

All studies were approved by the Human Research Ethics board (Pro00013094; Pro00001754) or the Animal ethic committee (AUP000002914) at the University of Alberta. All organ donors and their families provided written informed consent for research.

### Statistical analyses

Data analysis was performed using GraphPad Prism (v7.0c). Comparison of multiple groups was done by one‐ or two‐way ANOVA followed by Bonferroni or Tukey posttest. Data are expressed as means ± SEM, where *P* < 0.05 is considered significant. Associations between donor characteristics and insulin secretory responses were analyzed using Pearson correlation and GraphPad Prism (V.7), and the lines of best fit were obtained through linear regression.

## Results

### Autocrine activation of P2Y_1_ receptor induces protein kinase D1 phosphorylation on serine 916

**Figure 1:**
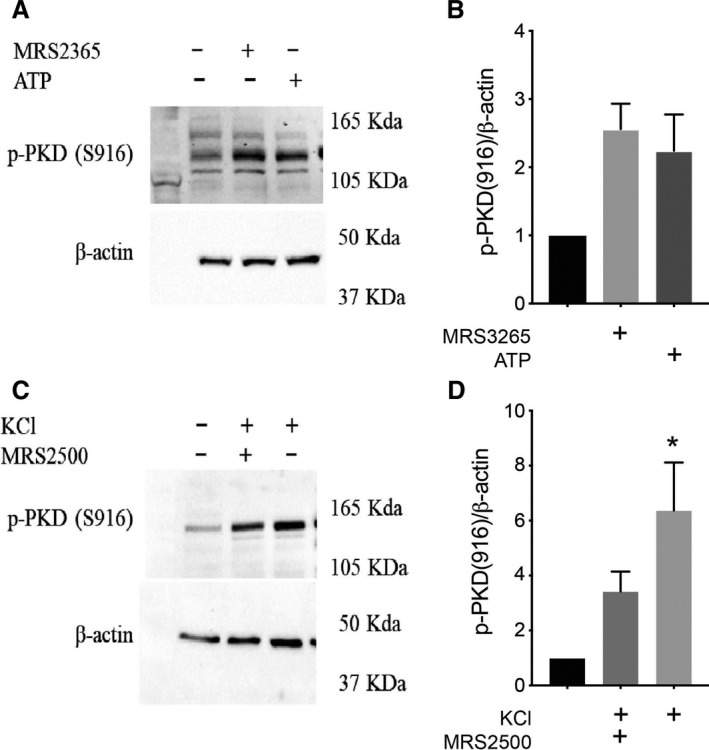
Effect of purinergic agonists and KCl‐induced depolarization on PKD1 activation. (A) Representative immunoblots of protein extracts from mouse islets stimulated for 20 min with the P2Y_1_ agonists, MRS2365 (100 nmol/L) or ATP (10 *μ*mol/L) at 1 mmol/L glucose and analyzed by Western blot for phospho‐PKD1 (serine 916) and *β*‐actin. (B) Quantification of blots from panel A. (C) Representative immunoblots of protein extracts from mouse islets stimulated for 20 min with KCl (30 mmol/L) ±P2Y_1_ antagonist, MRS2500 (1 *μ*mol/L) at 1‐mmol/L glucose and analyzed by Western blot for phospho‐PKD1 (serine 916) and *β*‐actin. (D) Quantification of blots from panel C. Data are mean ± SEM for three independent experiments, *P < 0.05 compared to the control condition.

Following a 20‐min exposure to P2Y_1_ receptor agonists, MRS2365 and ATP, PKD1 phosphorylation was induced at serine 916 in mouse islets (Fig. 1A and B), although this did not reach statistical significance by one‐way ANOVA (*P* = 0.055). In preliminary studies, we observed no impact of P2Y_1_ receptor activation on serine 744/748 phosphorylation (not shown) and did not focus further on this site. Direct depolarization with KCl caused significant induction of PKD1 phosphorylation and the effect was lost upon application of the P2Y_1_ antagonist, MRS2500 (Fig. 1C and D). Together, these data suggest that PKD1 phosphorylation on serine 916 is stimulated by P2Y_1_ receptor activation and that autocrine activation of the P2Y_1_ receptor induces this PKD1 phosphorylation in mouse islets. While it is unclear why we did not observe obvious serine 744/748 phosphorylation in our pilot studies, these results suggest that different pathways may have different regulatory actions on PKD1 since GPR4 ligands caused both serine 916 and serine 744/748 phosphorylation (Ferdaoussi et al. 2012). Antibodies against total PKD1 protein were found unreliable, and therefore, in these experiments, phospho‐PKD1 (serine 916) levels were normalized to *β*‐actin.

### P2Y_1_‐dependent potentiation of insulin secretion in mouse islets is mediated by protein kinase D1

**Figure 2:**
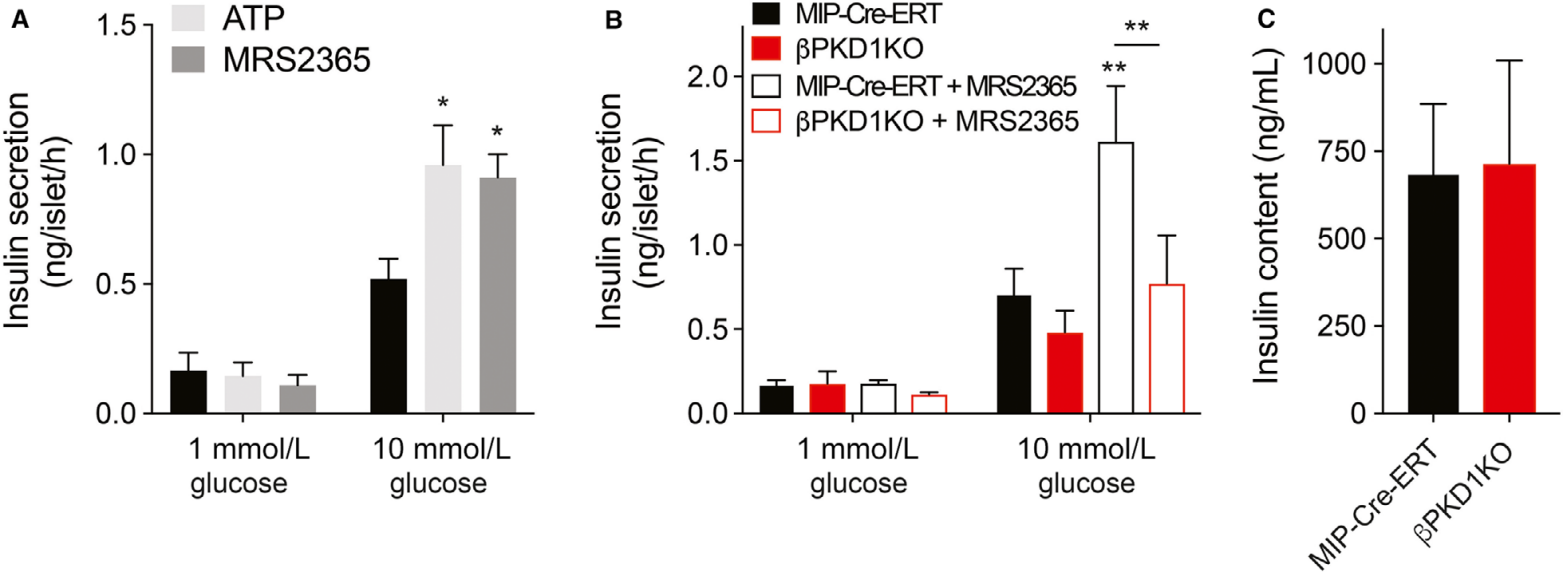
Potentiation of insulin secretion via activation of P2Y1 is PKD1 dependent. (A) Insulin secretion was measured from C57Bl/6 mouse islets over a 1‐h period at 1 and 10 mmol/L glucose in the presence of ATP (10 *µ*mol/L) or MRS2365 (100 nmol/L). (B) There was no significant difference in glucose‐stimulated insulin secretion between control MIP‐Cre‐ERT mice (black) and mice lacking *β* cell PKD1 (*β*PKD1KO; red), but the *β*PKD1KO failed to respond to the P2Y_1_ agonist, MRS2365 (100 nmol/L; open bars). (C) Insulin content assessed after the static incubations in MIP‐Cre‐ERT and *β*PKD1KO mouse islets was not different. Data are mean ± SEM for 3–5 independent experiments, *P < 0.05 and **P < 0.01 compared to the control condition or as indicated.

To examine the functional impact of PKD1 phosphorylation in response to P2Y_1_ agonists, its role in insulin secretion in C57Bl/6 and *β*PKD1KO mouse islets and MIP‐Cre‐ERT controls was investigated. There was significant potentiation of insulin secretion upon treatment with both P2Y_1_ agonists, ATP and MRS2365, at high glucose compared to the high‐glucose group alone in C57Bl/6 mouse islets (Fig. 2A). For the *β*PKD1KO and MIP‐Cre‐ERT mouse islets, no significant difference in insulin secretion was observed at high glucose between groups. However, upon addition of MRS2365 at high glucose, the MIP‐Cre‐ERT group had a dramatic potentiation of insulin secretion which the *β*PKD1KO group failed to mimic (Fig. 2B). This difference in secretion was not due to changes in insulin content (Fig. 2C). These results indicate that activation of P2Y_1_ potentiates insulin secretion from mouse islets in a PKD1‐dependent manner.

### P2Y_1_ increases *β* cell exocytosis in a protein kinase D1‐dependent manner

**Figure 3:**
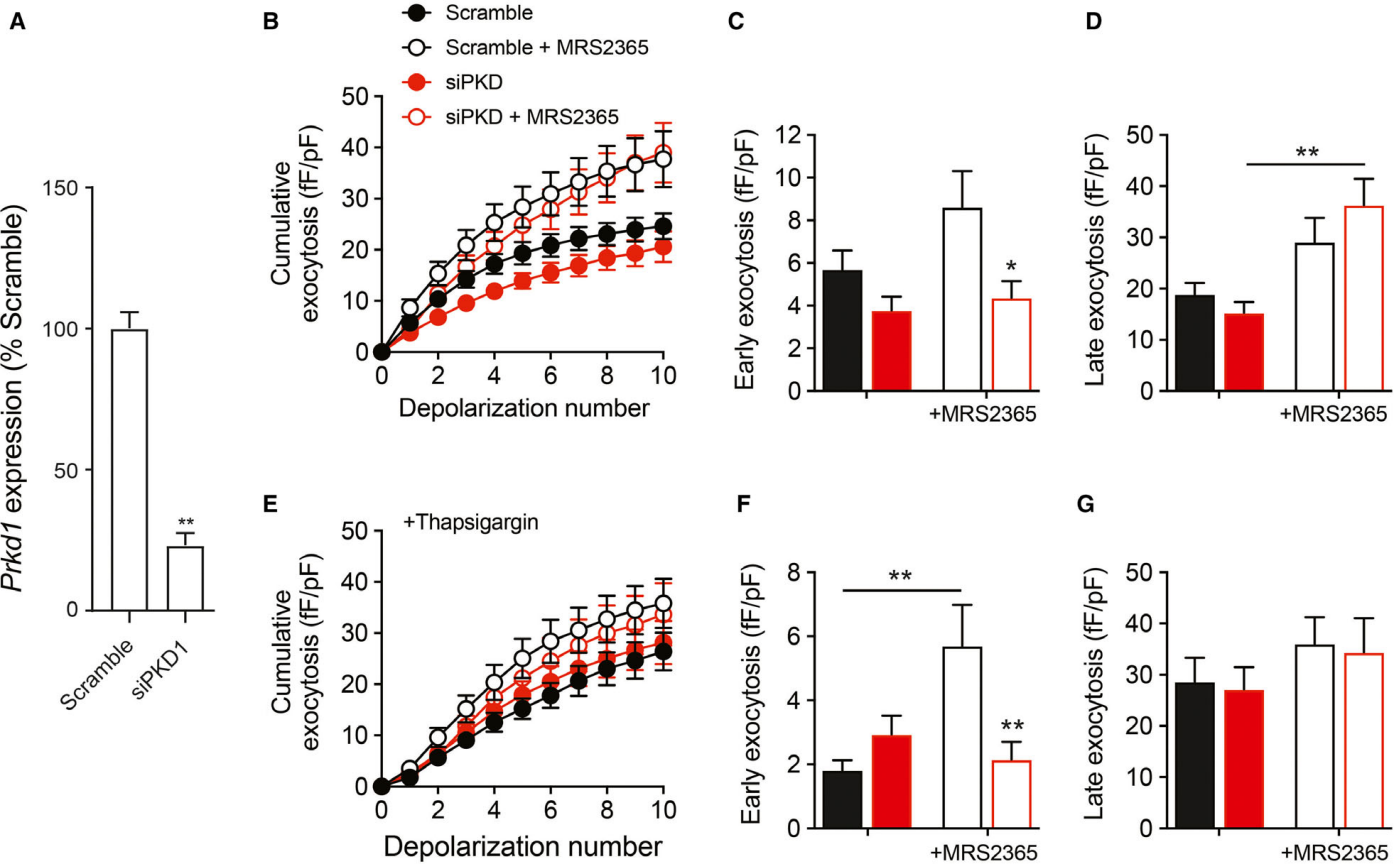
Effect of P2Y_1_ agonist on exocytosis and in single mouse *β* cells following PKD1 knockdown. (A) Transcript encoding PKD1 (*Prkd1*) was reduced in mouse islet cells transfected with siRNA targeting PKD1 (siPKD1) compared with a Scrambled control. In panels B–G, we measured exocytosis as membrane capacitance increases in response to a series of 10 membrane depolarizations by whole‐cell patch‐clamp of single *β* cells transfected with PKD1 siRNA (red) or scrambled control (black) and subsequently identified by insulin immunostaining. (B) The average cumulative capacitance responses in the presence or absence of MRS2365 (100 nmol/L). (C–D) The early exocytotic response (panel C; first depolarization) and late response (panel D; subsequent nine depolarizations) in the absence (closed bars) or the presence of MRS2365 (100 nmol/L; open bars). (E–G) The same as panels B–D, but following pretreatment with thapsigargin to deplete intracellular Ca^2+^ stores. Data are mean ± SEM for six experiments (panel A) or 14–20 cells in each group (panels B–G), *P < 0.05 and **P < 0.01 compared to the scrambled control or as indicated.

Next, the role for PKD1 in the P2Y_1_‐dependent facilitation of *β* cell exocytosis was examined. For this, siRNA‐mediated PKD1 knockdown was employed in mouse *β* cells. After 48 h, qPCR analyses of mouse *β* cells transfected with siRNA revealed a significant knockdown (87%) of PKD1 mRNA compared to scrambled control siRNA (Fig. 3A). To address whether *β* cell exocytosis is affected by PKD1‐knockdown, capacitance measurements of exocytosis were performed by whole‐cell patch‐clamp. A train of 10 depolarization steps from −70 to 0 mV evoked larger responses in control *β* cells in the presence of MRS2365 (Fig. 3A). While the exocytotic response to the initial depolarization (early exocytosis) is often taken to reflect exocytosis of a readily releasable pool of docked and primed granules, responses to subsequent depolarizations (late exocytosis) in part reflect refilling of this pool. In the absence of P2Y_1_ agonist there was no significant effect on the early exocytotic response (Fig. 3B–D). However, in response to the P2Y_1_ agonist both early and late exocytotic responses appear increased in control cells (Fig. 3B–D). The response of PKD1 knockdown cells to the P2Y_1_ agonist was blunted in the early phase exocytotic response (first depolarization; Fig. 3C) but not in the latter responses thought to reflect secretory vesicle recruitment (Fig. 3D). This suggests that the early exocytotic response, representing the readily releasable pool, is more dependent upon PKD1 following P2Y_1_ receptor activation than is the refilling of that granule pool.

Positive feedback signaling by ATP via the P2Y_1_ receptor leads to Ca^2+^‐induced Ca^2+^ release from endoplasmic reticulum stores which we showed previously to contribute to sustained *β* cell exocytosis (Khan et al. 2014). In a separate experiment, we pretreated cells with thapsigargin to deplete InsP_3_‐sensitive endoplasmic reticulum Ca^2+^ stores, and thus attempt to isolate the effects of P2Y1 agonism that are independent of intracellular stores. There was still no impact of PKD1 knockdown alone on exocytotic responses under this condition (Fig. 3E–G). And, although thapsigargin itself appears to reduce the early exocytotic response and decrease the late response (compare Fig. 3C with E and D with G), which could reflect the resulting perturbation of Ca^2+^ buffering, it should be noted that these are separate experiments (separate sets of mice and cells) and further work would be required to make a direct comparison. Following pretreatment of mouse *β* cells with thapsigargin, we found that MRS2365 could still facilitate the initial exocytotic response in a PKD1‐dependent manner (Fig. 3E and F). Activation of P2Y_1_ was not able to facilitate late (steps two to ten) exocytosis in cells pretreated with thapsigargin (Fig. 3G). These results indicate that P2Y_1_ potentiates exocytosis of the readily releasable pool of secretory granules in a PKD1‐dependent manner that is independent of intracellular Ca^2+^ stores, while effects on sustained exocytotic responses (i.e., refilling of the releasable pool) are likely more dependent upon Ca^2+^ release mediated by InsP_3_‐receptors and the PLC/DAG pathway as we reported previously (Khan et al. 2014).

### Potentiation of insulin through PKD1 correlates with glucose stimulation index and BMI of humans

**Figure 4:**
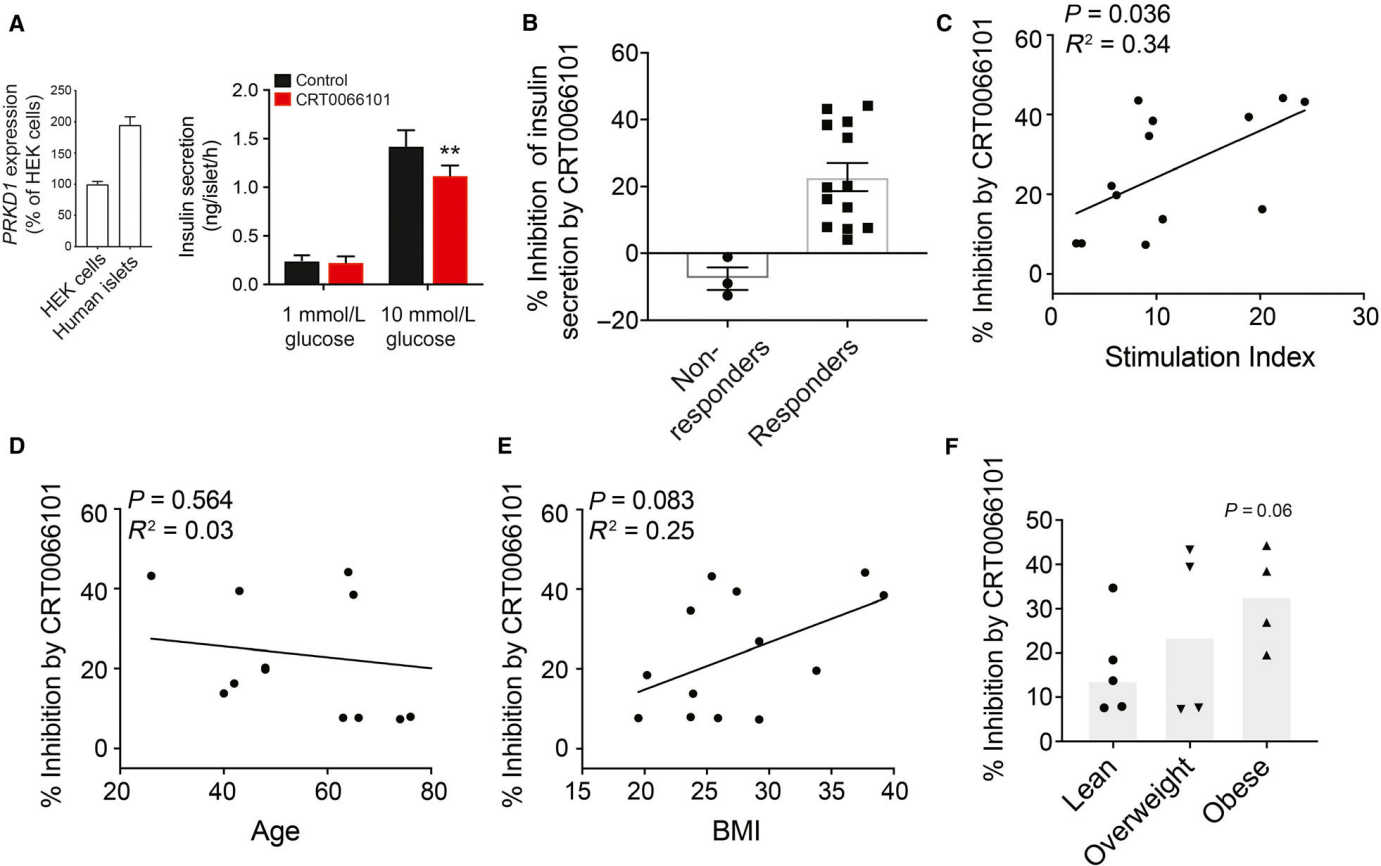
Ability of PKD1 to potentiate insulin secretion positively correlates with stimulation index and BMI of humans. (A) At right, we confirm the expression of transcript encoding PKD1 (*PKRD1*) in human islets, and at left we measure insulin secretion in (ng/islet/hr) in 1‐h static incubations in response to 1 and 10 mmol/L glucose in the presence of PKD1 inhibitor, CRT0066101 (10 *μ*mol/L). (B) Comparison of donor–donor response according to percentage inhibition of insulin secretion by PKD1 antagonist. (C–E) The percentage inhibition of responders after 20 min CRT0066101 treatment plotted against stimulation index (panel C; *n* = 13 donors), age (panel D; *n* = 13 donors), and BMI (kg/m^2^; panel E; *n* = 13 donors) in nondiabetic human islet donors. (F) Comparison of percentage inhibition by MRS2500 according to BMI tertiles (lean < 25; overweight 25–30; and obese> 35 kg/m^2^) (*n* = 5, 4, 4 donors).

To date, the role of PKD1 has not been investigated in human islets. Therefore, whether PKD1 is expressed in human pancreatic *β* cells or not and what might be the possible role of PKD1 in human islets were tested. In comparison with mice, where only *Prkd1* is expressed in islet cells (see https://tabula-muris.ds.czbiohub.org), human islets express transcripts for PKD2 and PKD3 at higher levels than they express PKD1. According to our own published RNAseq data (van de Bunt et al. 2015), accessible via www.t2dsystems.eu, expression of transcript encoding *PKRD1* in human islets (*n* = 118 donors) averages 2.2 transcripts per million (TPM) while *PRKD2* (27.4 TPM) and *PRKD3* (11.2 TPM) are more highly expressed. For this reason, a siRNA knockdown approach to a single isoform may not be advisable. We did, however, confirm the expression of *PRKD1* in human islets (Fig. 4A, inset).

**Figure 5:**
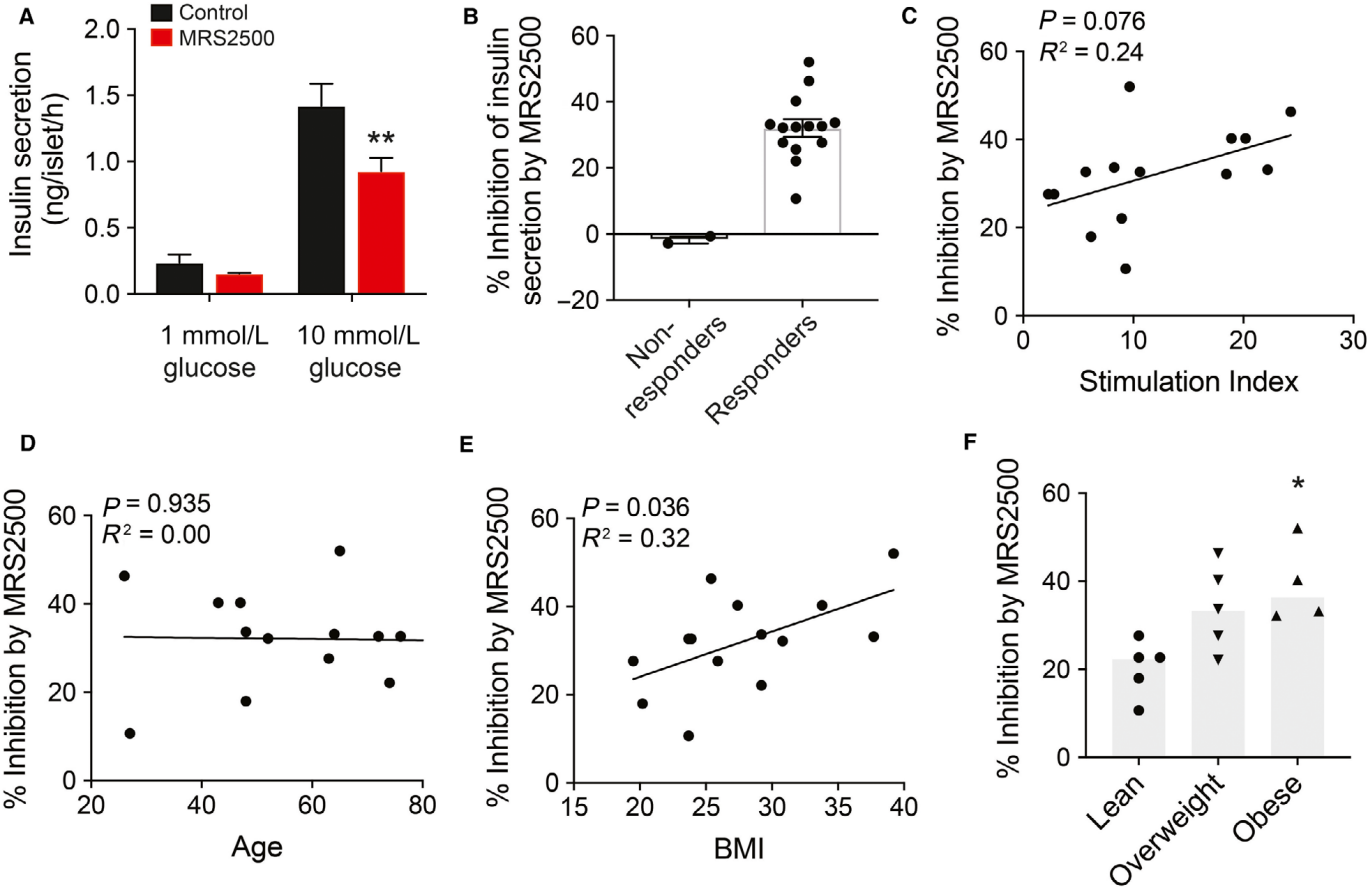
Ability of P2Y_1_ to potentiate insulin secretion positively correlates with BMI of humans. (A) Insulin secretion (ng/islet/h) was assessed in 1‐h static incubations in response to 1 and 10 mmol/L glucose in the presence of P2Y_1_ antagonist, MRS2500 (1 *μ*mol/L). (B) Comparison of donor–donor response according to percentage inhibition of insulin secretion by P2Y_1_ antagonist. (C–E) The percentage inhibition of responders after 20 min MRS2500 treatment plotted against stimulation index (panel C; *n* = 14 donors), age (panel D; *n* = 14 donors), and BMI (kg/m^2^; panel E; *n* = 14 donors) in nondiabetic human islet donors. (F) Comparison of percentage inhibition by MRS2500 according to BMI tertiles (lean < 25; overweight 25–30; and obese> 35 kg/m^2^) (*n* = 5, 5, 4 donors).

The ability of P2Y_1_ antagonist, MRS2500, and PKD inhibitor, CRT0066101 (IC_50_ 1–2.5 nmol for PKD1,2,3), to affect glucose‐stimulated insulin secretion was studied in islets from 16 human donors (Figs. 5 and 4). These reduced the secretory response to glucose by 35% and 20%, respectively (Figs. 5A and 4A). There was donor–donor variability in these responses. While the majority of donors showed reduced insulin secretion, there were 2–3 donors who quite clearly showed no response (Figs. 5B and 4B). There was no obvious difference between “responders” and “nonresponders” with respect to organ processing parameters (e.g., cold ischemia time, digestion time, etc.) or donor characteristics (age, sex, BMI, etc…), and it will likely require the assessment of many more donors to determine the reason for “nonresponsiveness” in a small number of donors. Within the majority of donors who showed reduced insulin secretion to these compounds, the inhibitory effect appears to correlate with stimulation index and donor BMI. In the responders, the percent inhibition of insulin secretion by P2Y_1_ antagonist tended to correlate with glucose stimulation index (*P* = 0.08; Fig. 5C), but not age (Fig. 5D), and was significantly correlated with donor BMI (*P* = 0.04, Fig. 5E). A significant difference in the percent inhibition of insulin secretion was also observed between lean and obese donors (Fig. 5F).

**Figure 6:**
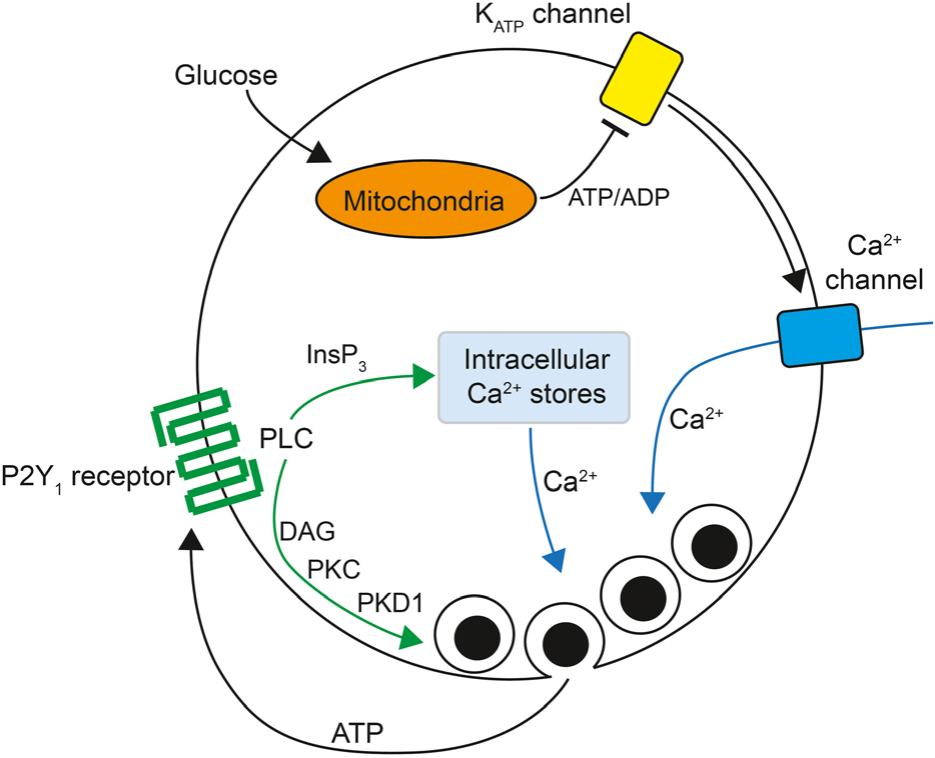
Proposed mechanism for P2Y_1_ receptor autocrine facilitation of insulin secretion. A glucose‐dependent generation of ATP results in closure of ATP‐sensitive K^+^ (K_ATP_) channels and membrane depolarization leading to activation of voltage‐dependent Ca^2+^ channels. The entry of Ca^2+^ promotes insulin granule fusion with the plasma membrane and release of insulin and other vesicular contents, including ATP. Subsequent activation of P2Y_1_ purinergic receptors activates phospholipase C (PLC) signaling and generation of inositol triphosphate (InsP_3_) and diacylglycerol (DAG). The former promotes a further increase in intracellular Ca^2+^ by promoting the release of intracellular stores and the latter acts to directly facilitate the exocytosis of releasable granules via a potential protein kinase C (PKC)‐dependent action on protein kinase D1 (PKD1). As noted in the text, an action via PKD1 may require synergistic pathways such as that mediated by the free fatty acid receptor GPR40.

For the PKD inhibitor, the percent inhibition also positively correlated with glucose stimulation index (*P* = 0.04; Fig. 4C), not with age (Fig. 4D), and tended to correlate with BMI (*P* = 0.08, Fig. 6E) for the responders. As with P2Y_1_, there may be a difference in the percent inhibition of insulin secretion when donors were separated into BMI tertials, PKD inhibition appears to be more effective in reducing insulin secretion from islets of obese donors (Fig. 4E), although this falls short of statistical significance. With both P2Y_1_ and PKD inhibition, we find no apparent correlation between inhibition to reduce insulin secretion and donor body weight, HbA1c, and sex. Thus, it appears that the P2Y_1_‐PKD axis plays a more prominent role in insulin secretion from nondiabetic donors with higher BMI and elevated secretory responses.

## Discussion

This study demonstrates that signaling via the P2Y_1_ receptor plays an important role in facilitating insulin release in part via PKD1 in pancreatic islets. An autocrine action is demonstrated as an increase in PKD1 phosphorylation upon depolarization of the islets which is inhibited in the presence of the P2Y_1_ antagonist. While we measured phosphorylation of serine 916 as a marker of PKD1 activity as has been reported previously (Matthews et al. 1999; Luiken et al. 2008; Sumara et al. 2009), this site may not always correlate with activity (Rybin et al. 2009), and other sites on PKD1 are often also used as indicators of activity – notably serine 744/748 (Matthews et al. 1999). In pilot experiments, we observed no phosphorylation of serine 744/748 in response to P2Y_1_ agonist (not shown), raising the possibility that P2Y_1_ activation may facilitate PKD1 activation by other pathways. Nonetheless, an important role for PKD1 downstream of P2Y_1_ activation is supported by our insulin secretion and electrophysiology studies following PKD1 inhibition, knockdown, or knockout.

The present data, in concert with our previously reported study (Khan et al. 2014), suggest a mechanism for P2Y1 facilitation of insulin secretion that involves two arms: an InsP_3_‐dependent increase in release of intracellular Ca^2+^ stores via Ca^2+^‐induced Ca^2+^ release; and a PKD1‐dependent pathway that may depend on DAG‐dependent PKC signaling (Fig. 6). Given results from the single‐cell exocytosis study, the former appears more important for refilling of granule pools during extended stimulation while the PKD1 component appears to facilitate exocytosis of the readily releasable pool. Indeed, PKD is known to prime vesicles for efficient transport and fusion to promote secretion of neurotensin (Li et al. 2004) and this requires the target protein Kidins220 which has been proposed to regulate more peripheral steps of exocytosis (Wazen et al. 2014). In line with this, another study observed twofold increase in membrane capacitance in the first few depolarizations in p38*δ*‐deficient pancreatic *β* cells compared to control *β* cells and attributed this increase in capacitance to an enhanced activity of PKD (Sumara et al. 2009). The results obtained here are compatible with this as we observe a PKD1‐dependent facilitation of exocytosis upon P2Y_1_ receptor activation that is limited to the first depolarizing step often thought to represent the fusion of granules from the readily releasable pool. It is difficult, however, to assign this impact of PKD1‐dependent increase in early exocytosis directly to bi‐phasic insulin secretion. However, it is possible that this pathway could increase first‐phase secretion via the early release of ATP mediated through pannexin channels (Bartley et al. 2019) or selective release from the granule fusion pore (MacDonald et al. 2006), or could promote increased second‐phase secretion via continued feedback during prolonged stimulation.

PKD1 affects various steps of glucose homeostasis. It was reported that PKD is involved in the transcriptional regulation of the insulin receptor gene (Zhang et al. 2010). Several studies corroborated its role in the trans‐Golgi network where it plays a key role in the formation and final cleavage of insulin granules (Baron and Malhotra, 2002; Gehart et al. 2012; Cruz‐Garcia et al. 2013). And finally, PKD1 is important for glucose‐stimulated insulin secretion as shown here and by others. We demonstrated previously that the free fatty acid (FFA), oleate, activates PKD1 through GPR40 which enhances insulin secretion (Ferdaoussi et al. 2012). It is well known that plasma FFA levels are elevated in obesity (Boden 2008). Elevated plasma FFA levels have been shown to account for insulin resistance in obese patients with type 2 diabetes (Boden, 2002). In light of the potential modulatory role for PKD1 phosphorylation on serine 916, it is therefore intriguing to speculate that the FFA receptor GPR40 and autocrine P2Y_1_ signaling may exert synergistic effects via PKD1 when FFAs are elevated in high‐fat diet (Bergeron et al. 2017) or human obesity. Accordingly, the magnitude of PKD1 inhibition of glucose‐stimulated insulin secretion observed is positively correlated with human donor BMI, where islets from lean human donors have minimal responses to PKD1 and maximal response to obese donors. We should note that these correlations were observed only when “nonresponding” human islet preps were removed. The exact reason why no inhibition of secretion with the P2Y_1_ or PKD1 antagonists was seen in these is unknown. These “nonresponders” were, however, relatively few (2–3 donors only, of 16 total) and it is possible that technical reasons underlie the lack of response to these agents. Additional donors will be required to determine this exactly; however, these results do fit within an in vivo study of the *β*PKD1KO mice, where we observed no significant differences in *β*PKD1KO and the control mice at basal condition but observed impaired glucose‐stimulated insulin secretion under high‐fat diet in *β*PKD1KO mice both in vivo and ex vivo (Bergeron et al. 2017).

A specific function of the PKD1 isoform of the PKD family with respect to autocrine purinergic signaling has not been elucidated so far. This is the first evidence that P2Y_1_/PKD1 pathway represents a mechanism of potentiation of glucose‐stimulated insulin secretion. Our data support a positive regulatory role for both P2Y_1_ and PKD1, particularly in obese (but nondiabetic) donors, suggesting a role for this pathway in compensatory upregulation of insulin secretion and that disruption of P2Y_1_‐PKD1 signaling may promote *β* cell dysfunction in type 2 diabetes.

## Conflict of Interest

The authors declare no conflict of interest relating to this work.
